# Single-Event-Upset Sensitivity Analysis on Low-Swing Drivers

**DOI:** 10.1155/2014/876435

**Published:** 2014-03-19

**Authors:** Nor Muzlifah Mahyuddin, Gordon Russell

**Affiliations:** ^1^School of Electrical and Electronic Engineering, Universiti Sains Malaysia, Engineering Campus, 14300 Nibong Tebal, Penang, Malaysia; ^2^School of Electrical, Electronic and Computer Engineering, Newcastle University, Newcastle upon Tyne NE1 7RU, UK

## Abstract

Technology scaling relies on reduced nodal capacitances and lower voltages in order to improve performance and power consumption, resulting in significant increase in layout density, thus making these submicron technologies more susceptible to soft errors. Previous analysis indicates a significant improvement in SEU tolerance of the driver when the bias current is injected into the circuit but results in increase of power dissipation. Subsequently, other alternatives are considered. The impact of transistor sizes and temperature on SEU tolerance is tested. Results indicate no significant changes in *Q*_crit_ when the effective transistor length is increased by 10%, but there is an improvement when high temperature and high bias currents are applied. However, this is due to other process parameters that are temperature dependent, which contribute to the sharp increase in *Q*_crit_. It is found that, with temperature, there is no clear factor that can justify the direct impact of temperature on the SEU tolerance. Thus, in order to improve the SEU tolerance, high bias currents are still considered to be the most effective method in improving the SEU sensitivity. However, good trade-off is required for the low-swing driver in order to meet the reliability target with minimal power overhead.

## 1. Introduction

In today's deep submicron, technology scaling relies on reduced nodal capacitances and lower voltages in order to improve performance and power consumption. This also includes shrinking the active chip area and increasing the layout density. Subsequently, this will reduce the critical charge required to upset a circuit node, making these submicron technologies more susceptible to soft errors. By having a very dense chip, the likelihood of having a large number of soft errors per chip is increasingly high. This is due to a higher susceptibility to alpha and neutron radiation [1–3]. Due to the increasing severity of the soft error problem, there is a growing trend in the community to adopt soft error rate as a design parameter along with the more common power, area, and speed trade-offs [4].

There have been a number of circuit level solutions and analysis methods proposed to address the issue with soft errors in logic designs [5–9]. These methods often rely on estimating key parameters and it is often that the impact of process variation is not included or considered irrelevant to the whole analysis. However, researches have shown that variations in technology parameters can lead to significant errors in the outcome, that is, the soft errors estimation. This could either become redundant in terms of over- or underestimate of the outcomes, or inaccurate modeling could affect the logic circuit main performance criteria.

There have been several distinct models to measure the effects of radiation on circuits at various technology nodes. Device level three-dimensional (3D) simulations using Technology Computer-Aided Design (TCAD) are very helpful for accurately predicting the behavior of these devices. Another approach is by using various SPICE-level current models [10, 11] to compute the critical charge metric. The shape and amplitude of the current model have a significant effect on the computation of the critical charge, *Q*_crit_. However, for this work, only SPICE-level circuit model is incorporated into the design as the test circuit only consists of 17 gates [12].

Traditionally, memories have been the most affected by SEU because small transistor sizes are used to increase memory density, resulting in lower capacitance and hence higher SERs [13]. However, memories can be protected by error detecting or correcting codes. Due to extensive technology scaling, it has been observed that unprotected combinational logic circuit is becoming more vulnerable to radiation-induced transient faults [14]. As it has been predicted, the SER in logic circuits per chip has become almost comparable to SER per chip of the memory elements [15].

It is known that low-swing driver performance can be affected by SEU as the driver's performance is mainly dependent on the switching activity at each node, carrying the intended low-swing across long interconnect. The SEU can alter the voltage swing by either increasing or decreasing the peak-to-peak voltage swing (Vpp). The effect is significant as both changes can influence the performance of the driver in terms of its robustness against noise and its propagation delay.

In order to ensure the signal integrity of the low-swing drivers, their reliability against any type of faults needs to be evaluated. Since radiation-induced faults such as an SEU have received significant attention in recent years, especially in deep submicron regime, it is important to investigate the performance of the low-swing driver against SEU effect using circuit design approach.

The circuit to be tested is a low-swing driver which incorporates diode-connected configuration at the output, or commonly known as the diode-connected driver. This type of driver has been studied in [12, 16], indicating high performance compared to other low-swing driver schemes. Therefore, a diode-connected driver known as the mLVSD driver [12] is chosen to be tested against SEU for this analysis as it has the best attributes among the diode-connected drivers.

Brief introduction on SEU as well as methods of measuring the component associated with SEU, that is, the critical charge, has been discussed in [17]. This work will be an extension of the paper where more details explanation on detecting the most sensitive nodes will be discussed. Subsequently, several additional approaches to improve the SEU tolerance will also be addressed, by incorporating the key parameters identified beforehand.

## 2. SEU: Background Review

**Figure 1:**
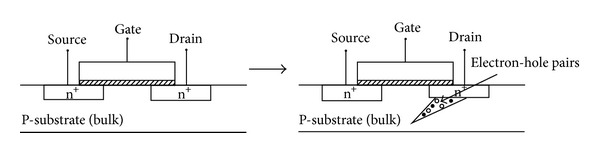
An illustration of how alpha particles strike a MOSFET device [8].

An SEU is a radiation-induced fault in an integrated circuit. The effect of an SEU is to change the behavior of the digital parts of a circuit in some unexpected manner, often producing incorrect results. When an energetic particle strikes a sensitive area such as the area near the reverse biased drain junction in a transistor, electron-hole pairs are generated, as shown in Figure 1. The amount of energy to create the electron-hole pairs is recorded at 3.6 eV for silicon, where, for energy of 1 MeV, the charge generated by a particle strike is 44.5 fC. Since a circuit node in 90 nm technology can store between 1 and 10 fC, a particle with energy of 1 MeV can alter the logic value stored on the node. This shows that, with every new technology node, circuit susceptibility to the effects of particle strikes increases. The minimum energy of a particle to create a voltage transition of sufficient strength to charge logic value on a node is given by

(1) $$\begin{array}{c} E_{\min}=3.6\times \frac{Q_{\text{crit}}}{q}, \end{array}$$

where *Q*_crit_, critical charge, is the amount of charge necessary to trigger a change in the logical level.

## 3. Measurement and Modeling of an SEU

Modeling of an SEU at the circuit level is commonly done using a current source at the impacted node and a measurement of *Q*_crit_. *Q*_crit_ is an important parameter in measuring the SEU sensitivity of a circuit node [18]. In order to measure *Q*_crit_, a current source is used to model the current pulse created by the ion strike. The current source is modeled in the form of double exponential waveform [19] described as

(2) $$\begin{array}{c} I\left(t\right)=\frac{Q_{\text{crit}}}{\left(\tau_{F}-\tau_{R}\right)}\left[\exp\left(\frac{-t}{\tau_{F}}\right)-\exp\left(\frac{-t}{\tau_{R}}\right)\right]. \end{array}$$

This is the most commonly used model where the two timing parameters (*τ*_*R*_ and *τ*_*F*_) represent the rising and falling time constants of the exponentials. This model has been widely used in the literature to find not only the *Q*_crit_ but also the SEU introduced by ion strikes in combinational logic [20].

There are few required steps in order to measure the *Q*_crit_ for SEU tolerance analysis. These steps are generally used for fault injection experiment, which cover three processes [21].

*Fault Target Location.* The typical targets of model-based fault injection techniques are located at ports, signals, nodes, and variables.

*Fault Injection.* This involves provoking the occurrence of a fault in the circuit by reconfiguring the internal resources. In this case, the bias current will be injected varying from 0.01 to 0.5 mA which significantly will alter the process parameters of the circuit.

*Observation of Fault Consequences*. Once the fault has been injected, it is necessary to observe how the system reacts. Usually a trace of the outputs and the state of the system is stored for its interior analysis. Based on this work, the driver might react by changing its low-swing characteristics or stop functioning as a low-swing driver, when one of more different parameters is changed during the current injection.

Based on the steps listed earlier, the most sensitive nodes on the driver need to be located, in order to place the current source; thus all nodes on the driver were tested against the SEU, by observing the changes in the output voltage swing. Each node on the driver is tested one by one by injecting the current source modeled by (2). Changes in the voltage swing of the driver are monitored as the current is increased from 0.01 mA to 0.5 mA.

**Figure 2:**
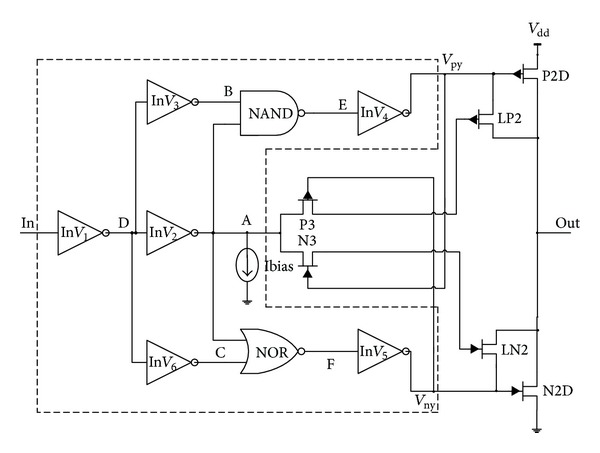
Circuit implementation of SEU analysis.

**Figure 3:**
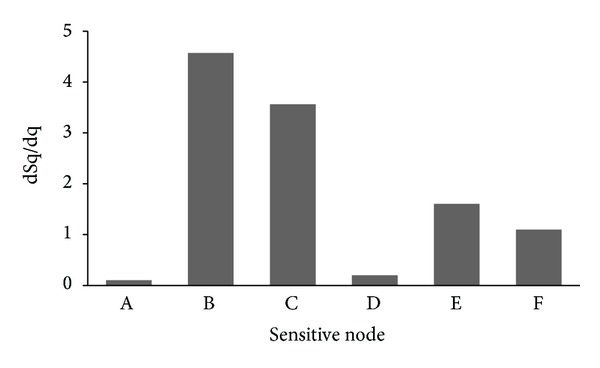
Sensitivity measurement of nodes on driver circuit.

The most sensitive nodes are not only referred to the nodes between logic gates but also implied on the nodes within the gates too. Preliminary results show that the most sensitive nodes in these circuits are located in the digital part of the driver; the box area of the circuit is shown in Figure 2. These nodes labeled A to F are among the most sensitive nodes in the circuit. The result shown in Figure 3 shows that node A has the lowest dSq/dq indicating the smallest critical charge compared to other nodes, meaning that node A is most susceptible to soft errors. In this instance, a current source is placed at node A, which is the most sensitive node.

The sensitivity towards SEU is measured in terms of *Q*_crit_ by varying the bias current. All measurements of *Q*_crit_ are from HSPICE simulations using the 90 nm technology. The impact of different parameters such as transistor sizes or W/L ratio and operating temperature on the SEU tolerance of the driver is discussed.

### 3.1. Parameter Screening

The methods implemented in [22] suggested the use of screening experiment based on statistical approach, that is, the Plackett Burman approach. The method can identify and estimate the effect of key factors towards SEU tolerance. This is carried out through measurement of *Q*_crit_ for various CMOS logical cells using HSPICE simulation. The statistical technique can identify the error in *Q*_crit_, SEU, and SER measurements which are likely to occur in the simulations. Subsequently, the statistical outcomes can support the accuracy of the reliability estimation, as well as identify additional properties of *Q*_crit_ measurements at the circuit level. In [22] several technology parameters have been identified and grouped into larger categories such as voltages, parameter sizing, current injection models, and operating parameters.

The voltage supply, *V*_dd_, was considered under voltage category as well as threshold voltage, Vth. *V*_dd_ has been identified as a critical parameter in most studies. It is known that *Q*_crit_ scales together with *V*_dd_ due to the approximation relationship of *Q* = CV, where *Q* responds to *Q*_crit_ and V is *V*_dd_. This scaling trend can be seen in [23]. Subsequently, *V*th is also considered to be one of the key parameters due to its impact on leakage current and consequently on SEU and *Q*_crit_ [6, 7, 18, 24, 25]. Similar to *V*_dd_, Vth also changes with *Q*_crit_, as an increase in *V*th will result in the increase in SEU attenuation [6].

Besides voltages, parameter sizing, specifically transistor sizing, is often identified as a critical parameter. Parameter sizing has a direct impact on the drive current and consequently on *Q*_crit_. The following parameters were identified in the previous studies: minimum channel length, Lmin; minimum width to length ratio, Wmin/Lmin; PMOS width to NMOS width, Wp/Wn; minimum diffusion length [6, 8, 24, 26–29].

Aside from voltages and parameter sizing, the current source model was also considered. It comprises *Q*_crit_, charge collection, and establishment time which can be represented by the bandwidth of the current pulse modeled using an ideal current source, as a standard practice for circuit level simulation [29–31].

In addition, the operating condition of the circuit was also included in the analysis in which the operating temperature is chosen as it has significant impact on the mobility and carrier concentration of the charges, hence, directly affecting *Q*_crit_.

Within these categories, several parameters were identified and analyzed in [22]. The statistical analysis indicates that when values are unknown and with large variation, these parameters have the largest effect on *Q*_crit_: Wmin/Lmin and *V*_dd_. Large variation in other parameters appears to have secondary effects on *Q*_crit_, that is, the current model, the operating frequency, and temperature. Hence, these final parameters are implemented in the SEU analysis in order to acquire the most effective method in improving SEU tolerance.

### 3.2. Preliminary SEU Analysis

From the SEU tolerance analysis in [13], several methods have been introduced in order to mitigate the problems with SEU. These methods are by introducing a high bias current increasing *V*_dd_ or the operating frequency. These parameters are some of the key parameters identified previously, which have the most impact on SEU sensitivity. The analysis starts with the impact of bias current on the SEU tolerance, tested at temperature of 25°C, varying *V*_dd_ from 0.6 V to 1.0 V and operating frequency from 0.5 GHz to 1.0 GHz. The result indicated that *Q*_crit_ increases with the bias current. This means that the effect of SEU on the circuit can be minimized when larger bias current is applied. This is because larger current implies larger transistor size and as a result larger capacitances to hold charges and hence greater immunity to SEU.

Another method was also applied which is by increasing voltage swing or in this case the voltage supply. It is found that the *Q*_crit_ increases with *V*_dd_ due to better margin on the effective voltage of the diode-connected transistors at the output. This in turn will also increase the voltage swing. With higher voltage swing, more charge is needed to upset the affected node.

Last method was tested against the operating frequency where the result indicated that SEU tolerance works better at high frequency. At higher frequencies, the ionization time constants are independent of the operating frequency; thus at some points they will become larger than the operating frequency. Thus, more energy is required for an SEU to have an effect in the short period of time, which makes the circuit less sensitive at higher frequency.

In terms of SEU tolerance, the results in [13] which are qualitatively compared in Table 1 indicate that by introducing high bias current, SEU tolerance is improved significantly but with the cost of power consumption. Other two methods can be used, but the improvement is less significant than employing high bias current. Hence, a good trade-off between two design parameters was suggested in the end.

Subsequently, there are other possibilities that can be tested before succumbing to earlier conclusive judgment, by analyzing the impact of temperature and transistor sizes. The impact of transistor sizes on SEU tolerance of the driver is tested at fixed temperature of 300 K, *V*_dd_ of 1 V, and operating frequency of 1 GHz whilst injecting bias current varying from 0.03 to 0.5 mA. Meanwhile the impact of the operating temperature is tested at fixed *V*_dd_ of 1.0 V and operating frequency of 1 GHz, whilst varying the temperature from 300 K to 398 K, at different values of bias currents.

## 4. Results and Discussion

**Figure 4:**
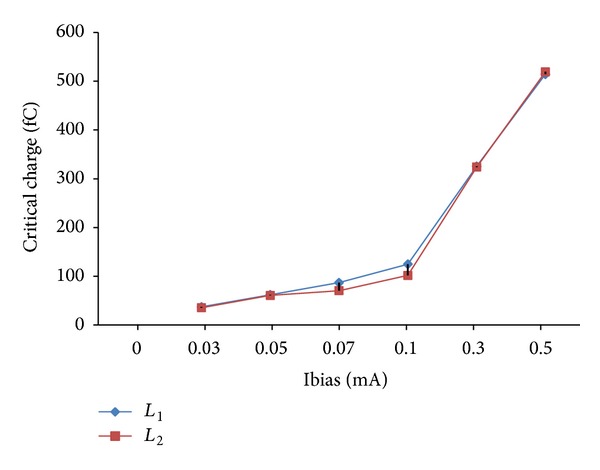
Critical charge against bias current for different Leff at 1.0 V.

Figure 4 indicates that by increasing the W/L ratio or choosing larger effective transistor length (Leff), there is no effect towards *Q*_crit_ as the bias current is increased from 0.03 to 0.5 mA. The Leff is increased by 10% from the nominal value of 90 nm. In addition, from Table 2, the amount of increase in *Q*_crit_ is shown for different values of Leff at an operating frequency of 1 GHz. It can be seen that the value of *Q*_crit_ increases from 14 to 15 times as the bias is increased from 0.03 to 0.5 mA at Leff of 90 (L1) and 99 nm (L2), respectively. There is only 5% increase in *Q*_crit_ between L1 and L2. This indicates that the rises in *Q*_crit_ depend solely on the increase in the bias current regardless of the effective transistor length that is being used. However, different results might be acquired when different technology nodes are used.

**Figure 5:**
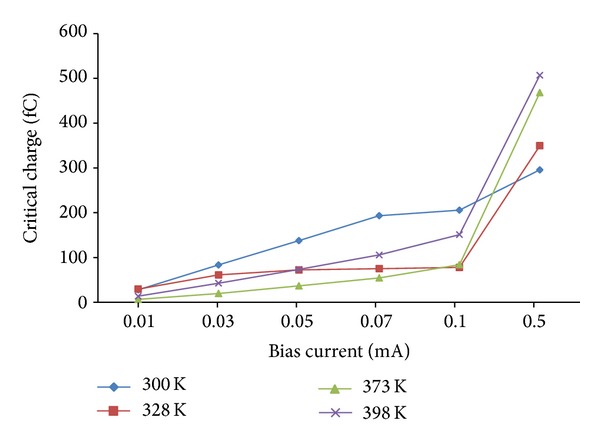
Critical charge against bias currents for different operating temperature at 0.5 GHz and *V*_dd_ of 1.0 V.

Another method in improving SEU tolerance is by increasing the operating temperature. The mLVSD driver is simulated at the normal temperatures of 300 K, 328 K, 373 K, and 398 K, varying the bias current from 0.01 to 0.5 mA.  Figure 5 indicates a gradual increase in *Q*_crit_ from 0.01 to 0.1 mA for all temperatures. However, as the temperature reaches 0.5 mA, there is a sharp increase (~12 times) for all three temperatures, that is, 328 K, 373 K, and 398 K.

Table 3 reflects on this increase for 328 K and above. This means that a significant improvement on SEU tolerance can be achieved when a very high temperature at high bias currents is used. However, at 300 K there is very little significant change or pattern in *Q*_crit_ indicating that the temperature has less effect on SEU tolerance for temperature less than 300 K [14]. It is known that there will be inaccuracies in the model at low temperature due to the noise exhibited in this region [32].

Subsequently, it can be decided that the sharp increase in *Q*_crit_ at temperature beyond 328 K may be due to the effect of device process parameters at high temperature, such as the funneling length and depletion region width [33]; thus their temperature dependence also depends on the doping level as well as the ratio between the mobility of holes and electrons. Subsequently, few studies also state that the SEU tolerance can be improved at higher temperature [34, 35], whilst study in [32] illustrates how the collected charge, which is also a component of *Q*_crit_, linearly increases with temperature, as shown in the Klien model.

## 5. Conclusions

In terms of SEU tolerance, previous results indicate that, although by introducing a high bias current into the design which will improve the reliability towards SEUs, the power consumption is significantly increased. For this work, other alternatives, that is, by increasing the effective transistor length and temperature, are discussed. One method of increasing W/L ratio or choosing larger effective transistor length shows that the improvement is less significant than employing high bias current. The Leff is only increased by 10% as larger percentage increase will totally change the device operation.

Subsequently, the SPICE simulation and the analytical modeling show that, depending on the device, temperature can have an effect on the SEU tolerance. However, the temperature itself consists of several subparameters that can contribute to the effect on SEU sensitivity such as the depletion width or diffusion length of a transistor. Subsequently, many process parameters are strongly temperature dependent; thus it is difficult to emphasize the direct impact of temperature on the SEU tolerance without considering other important parameters. One effective solution that can be implemented is by performing parameter screening for only the temperature dependent parameters, in order to identify the key parameters for this category. This can be easily carried out using the Plackett Burman approach.

However for this paper, in order to improve the SEU tolerance, the high bias currents are considered the most effective method compared to *V*_dd_, operating frequency, temperature, and transistor size in improving the SEU tolerance of this driver circuit. However, a good trade-off is required in order to meet the reliability target with minimal power overhead.

## Figures and Tables

**Table 1:** Qualitative comparison of *V*_dd_, current source, and frequency against *Q*_crit_.

Parameter setting	Parameters	Increase in *Q*_crit_
30–500 *μ*A	*I*_bias_	x1.45
0.8–1.0 V	*V*_dd_	x3.55
1.0-2.0 GHz	Frequency	x1.15

**Table 2:** *Q*_crit_ improvement with current bias for different effective length at 0.5 GHz.

Leff (nm)	*Q*_crit_ (fC) at *I*_bias_ = 30 uA	*Q*_crit_ (fC) at *I*_bias_ = 500 uA	Increase in *Q*_crit_
L1 (90)	37.26	514.3	x13.79
L2 (99)	35.6	519.3	x14.59

**Table 3:** *Q*_crit_ improvement with temperature for different bias currents at *V*_dd_ of 1.0 V.

Bias current (*μ*A)	*Q*_crit_ (fC) at −300 K	*Q*_crit_ (fC) at 398 K	Increase in *Q*_crit_
30	83.43	42.55	X0.5
50	137.65	73.28	X0.53
500	295.78	507.09	X1.71

	X3.54	X11.9	X3.36
